# Influence of Cu on the Improvement of Magnetic Properties and Structure of *L*1_0_ FePt Nanoparticles

**DOI:** 10.3390/nano11051097

**Published:** 2021-04-23

**Authors:** Luran Zhang, Xinchen Du, Hongjie Lu, Dandan Gao, Huan Liu, Qilong Lin, Yongze Cao, Jiyang Xie, Wanbiao Hu

**Affiliations:** 1 National Center for International Research on Photoelectric and Energy Materials, School of Materials and Energy, Yunnan University, Kunming 650091, China; dxc1134@163.com (X.D.); yunnanhaoguoduo@gmail.com (H.L.); cake_1124@mail.ynu.edu.cn (D.G.); liuhuan0741@126.com (H.L.); lin_qilong@outlook.com (Q.L.); 2 Department of Physics, Dalian Maritime University, Dalian 116026, China; cyz@dlmu.edu.cn

**Keywords:** FePt nanoparticles, magnetic materials, coercivity

## Abstract

*L*1_0_ ordered FePt and FePtCu nanoparticles (NPs) with a good dispersion were successfully fabricated by a simple, green, one-step solid-phase reduction method. Fe (acac)_3_, Pt (acac)_2_, and CuO as the precursors were dispersed in NaCl and annealed at different temperatures with an H_2_-containing atmosphere. As the annealing temperature increased, the chemical order parameter (*S*), average particle size (*D*), coercivity (*H*_c_), and saturation magnetization (*M*_s_) of FePt and FePtCu NPs increased and the size distribution range of the particles became wider. The ordered degree, *D*, *H*_c_, and *M*_s_ of FePt NPs were greatly improved by adding 5% Cu. The highest *S*, *D*, *H*_c_, and *M*_s_ were obtained when FePtCu NPs annealed at 750 °C, which were 0.91, 4.87 nm, 12,200 Oe, and 23.38 emu/g, respectively. The structure and magnetic properties of FePt and FePtCu NPs at different annealing temperatures were investigated and the formation mechanism of FePt and FePtCu NPs were discussed in detail.

## 1. Introduction

Magnetic nanomaterials, one kind of the most important functional materials, have been widely used in many fields. Among various magnetic nanomaterials, chemically ordered *L*1_0_-FePt has attracted much attention because of its very high magnetocrystalline anisotropy constant (*K*_u_) (7 × 10^7^ ergs/cc), which allows very low critical superparamagnetic size (~ 3–4 nm), high Curie temperature, good chemical stability, and biological compatibility [1,2,3]. These properties promote the important potential applications of *L*1_0_-FePt in the high-density magnetic recording medium [4,5], high performance permanent magnetic materials [6,7], catalysts [8,9], and biological applications [10,11,12,13].

In 2000, Sun et al. successfully prepared mono-disperse spherical FePt nanoparticles (NPs) by using a thermal decomposition method [14]. Fe (CO)_5_ and Pt (acac)_2_ were used as Fe and Pt sources, respectively, to decompose Fe (CO)_5_ at high temperature (297 °C) in a dioctyl ether solvent containing surfactant of oleic acid and oleylamine and to reduce Pt (acac)_2_ with hexadecanediol to obtain FePt nanoparticles of face-center cubic (FCC) phase. Since the pioneering work of Sun et al., the size and morphology control of FePt NPs have been extensively studied. FePt nanorods [15,16], nanowires [17,18], nano-cubes [19], and coral-shaped [20,21] NPs of the fcc phase have been successfully prepared. However, FePt NPs with fcc phase show low magneto-crystal anisotropy and soft magnetic or superparamagnetic at room temperature which greatly limits their applications. In general, in order to obtain *L*1_0_-FePt, samples need to be heat treated above 500 °C, which causes undesirable agglomeration and sintering of FePt NPs. For preventing sintering and agglomeration during the annealing process, coating fcc-FePt NPs with a layer of high melting point material (MgO or SiO_2_) was proposed [22,23]. This method requires multi-step operations: firstly, fcc-FePt NPs are synthesized by the liquid phase method, then coating fcc-FePt NPs with MgO or SiO_2_, finally carrying out heat treatment and shell removal. Subsequently, heat treatment of the prepared fcc-FePt NPs in salt baths was proposed. All of these methods require multiple steps to prepare *L*1_0_-FePt NPs. In addition, reducing the ordering temperature of FePt is also an option to prevent FePt NPs from agglomeration and sintering. The ordering temperature of FePt can be reduced by adding about Cu, Ag, or Au elements [24,25]. Wang et al. also reported that the addition of 29% Ag could reduce the ordering temperature of FePt nanoparticles to 400 °C [26]. However, the proportion of *L*1_0_-FePt fabricated by this method was low and the coercivity was only 7600 Oe. Moreover, a large amount of Ag would greatly reduce the saturation magnetization of the sample.

In this study, a simple, green, and high-yield one-step solid-phase reduction method for *L*1_0_-FePt NPs was proposed. In this method, the precursors of Fe (acac)_3_ and Pt (acac)_2_ were directly mixed with NaCl by ball milling and then annealed at certain temperatures. Since Fe (CO)_5_ produced toxic CO gas during thermal decomposition, Fe (acac)_3_ was used as the Fe source in this study. In the study, NaCl was not only used as a substrate when FePt NPs are generated but also as a separating material to prevent FePt NPs from agglomeration and sintering. This method does not require any organic solvents, surfactants, chelators or catalysts. The composition of FePt NPs can be easily adjusted by changing the ratio of precursors. The magnetic properties of FePt NPs can be adjusted by controlling the annealing temperature. Besides, 5% CuO was added to the precursors, which successfully reduced the FePt ordering temperature and improved the magnetic properties of FePt NPs. The structure, magnetic properties, and formation mechanism of FePt and FePtCu NPs at different heat treatment temperatures have been investigated and discussed.

## 2. Materials and Methods

In this work, the precursors, Fe (III) acetylacetonate (Fe (acac)_3_ 99.9%) and Pt (II) acetylacetonate (Pt (acac)_2_ 98%), were provided by Aladdin, Shanghai, China. CuO (99%) was purchased from Alfa Aesar, Shanghai, China. NaCl (99.5%) was provided by Aladdin, Shanghai, China, and was dried in an oven at 80 °C for 4 h before used. All the reagents were used without further purification. For preparing the *L*1_0_-FePt nanoparticles, NaCl was ball milled for 12 h and then Pt (acac)_2_ and Fe (acac)_3_ at the mole ratio of 1:1 were mixed with NaCl and ball milled for another 24 h. The weight ratio of the precursors and salt was fixed at 1:1000. In this work, all samples were ball milled in one batch (FePt in 2 ball milled pots, FePtCu in another 2). Then, the powder was put into arks and annealed at 400, 500, 550, 600, 650, 700 and 750 °C for 2 h in a tube furnace under a 5% H_2_ and 95% Ar mixed gas atmosphere. The flow rate of gas was kept at 40 sccm. The heating rate was 10 °C/min. At each annealing temperature, two arks (one was for FePt series, another was for FePtCu series) were put in the furnace. After annealing, the samples were cooled down to room temperature in the reducing gas atmosphere and then were washed with de-ionized water for several times to remove NaCl. The preparation method of FePtCu NPs was the same as that of *L*1_0_-FePt NPs, which just added an extra 5% CuO in the precursors.

A vibrating sample magnetometer (VSM) in the physical property measurement system (PPMS) was utilized to measure the magnetic properties of the samples at 300 K. The electron probe x-ray microanalysis (EPMA) was utilized to determine the elemental compositions of the samples. The structural analysis was performed by X-ray diffraction (XRD) with Cu K_α_ radiation (SmartLab^TM^ X, Rigaku Corporation, Tokyo, Japan). The morphology and the lattice fringes of nanoparticles were characterized by transmission electron microscope (TEM)(JME-2100, JEOL Ltd., Tokyo, Japan). For TEM observation, the powder sample was dispersed in alcohol and then dropped onto a carbon film supported on a copper grid.

## 3. Results

**Figure 1:**
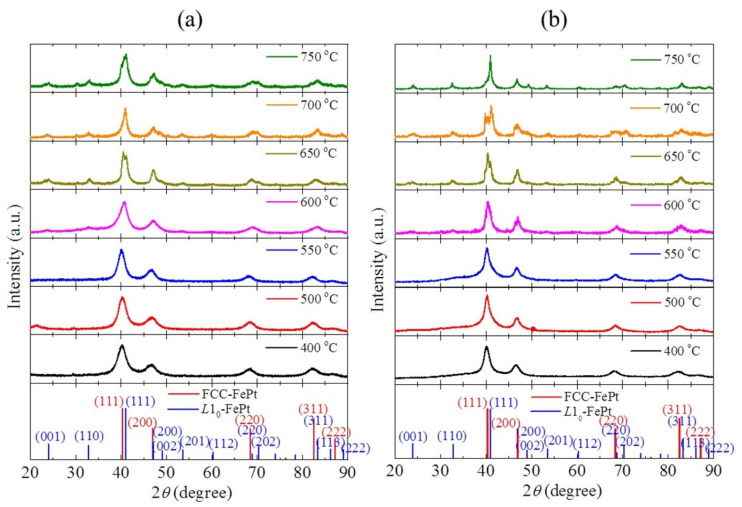
XRD patterns of (**a**) FePt and (**b**) FePtCu nanoparticles at different annealing temperatures.

Figure 1 shows the XRD patterns of FePt and FePtCu nanoparticles at different annealing temperatures. It can be seen that the FePt NPs prepared at 400 °C shows the fcc structure. The (111), (200), (220), and (311) peaks appear but the characteristic peaks for *L*1_0_-FePt, such as (001), (110), and (002) peaks, are not found. With the temperature increases, the peaks shift to the high angle which means that FePt NPs started to form the *L*1_0_ phase. When the temperature increases to 600 °C, the superlattice peaks (001) and (110) show up. With the further increase of the temperature, (002), (201), (112), and (202) peaks that belonged to *L*1_0_-FePt are detected. It should be noticed that when the temperature is higher than 650 °C, a bimodal phenomenon occurs at the (111) peak, which means that both the fcc phase and the *L*1_0_ phase of FePt exist in the sample even when the temperature reached 750 °C. FePtCu NPs are similar to FePt NPs. It should be noticed that FePtCu NPs that annealed at 550 °C have a weak peak corresponding to (110), which is almost undetectable. When the temperature reaches 750 °C, the bimodal phenomenon disappears and the (200) and (002) peaks are clearly separated, which suggests the higher ratio of the *L*1_0_ phase. For both FePt and FePtCu samples, the higher the annealing temperature, the stronger the observed characteristic peaks of the *L*1_0_ phase.

**Figure 2:**
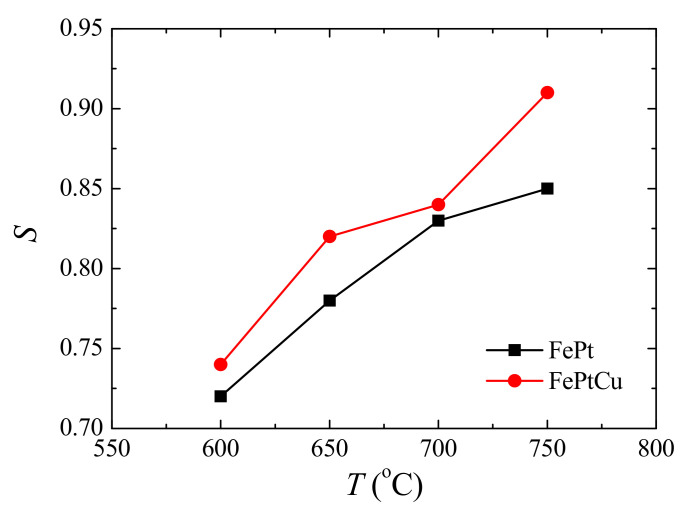
Chemical order parameter (*S*) of FePt and FePtCu nanoparticles at different annealing temperatures.

The ordered degree of FePt can be reflected by the chemical order parameter (S) [27]. The *S* can be calculated from the following equation [28]: $S\approx 0.85{\left(\frac{I_{001}}{I_{002}}\right)}^{\frac{1}{2}}$, where *I*_001_ and *I*_002_ are the intensities of the (001) and (002) peaks. Figure 2 shows the *S* of FePt and FePtCu NPs at different annealing temperatures. For both FePt and FePtCu NPs, *S* increases with the increasing annealing temperature. *S* of FePt NPs increases from 0.72 to 0.85 when the annealing temperature increases from 600 to 750 °C, and that of FePtCu NPs increases from 0.74 to 0.91. It can be found that the FePtCu NPs have higher *S* than that of FePt NPs at the same temperature, which means that FePtCu needs a lower annealing temperature to reach the same ordered degree. *S* of FePtCu NPs annealed at 750 °C can reach 0.91. The improved *S* and the reduction of the ordering temperature of FePt alloying by adding Cu also have been proved by other works [24,25].

**Figure 3:**
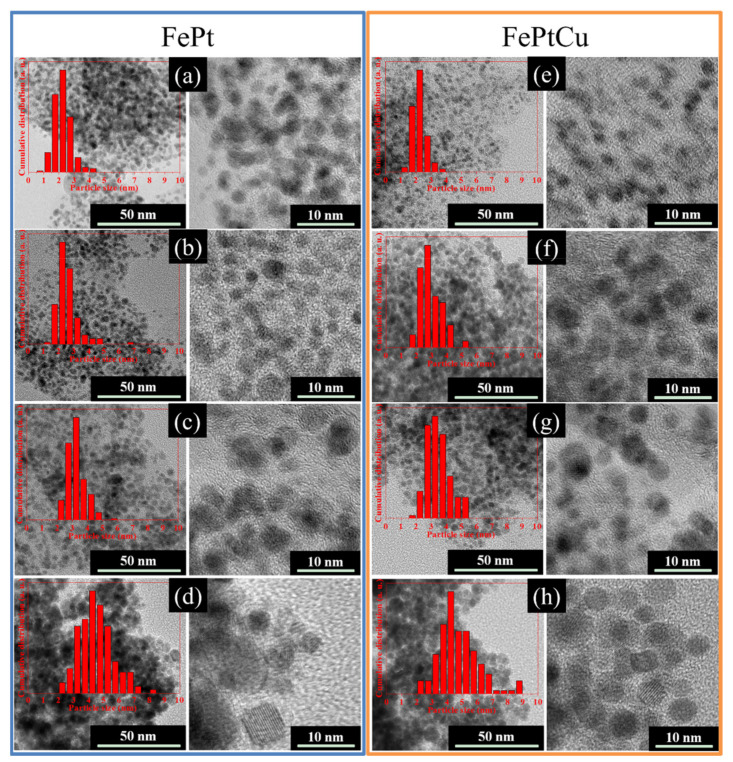
TEM and HRTEM images of the FePt nanoparticles(NPs) annealed at (**a**) 400 °C, (**b**) 550 °C, (**c**) 650 °C, and (**d**) 750 °C and FePtCu NPs obtained at (**e**) 400 °C, (**f**) 550 °C, (**g**) 650 °C, and (**h**) 750 °C. The insets are the size distribution of the particles.

TEM and HRTEM images of the FePt and FePtCu NPs annealed at different temperatures are shown in Figure 3. The samples were dispersed in alcohol and dropped onto a carbon film supported on a copper grid to test, no surfactant was used; therefore, the particles look like little aggregates. From the HRTEM images, the particles show a good dispersion that no sintering or coalescence was observed which means that NaCl can effectively prevent FePt from coalescing or sintering during the annealing process. The size distributions of the particles are shown in Figure 3 (insert images). In each case, over 200 particles were counted to determine the particle size and particle size dispersion.

**Figure 4:**
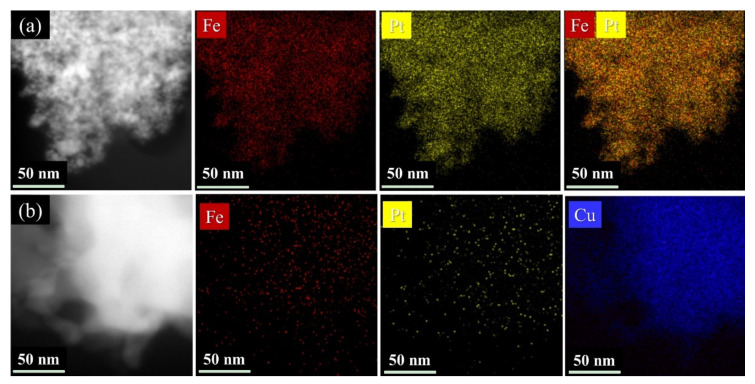
Scanning transmission electron microscopy - energy dispersive X-ray spectroscopy(STEM-EDS) element mapping of (**a**) FePt and (**b**) FePtCu NPs synthesized at 750 °C.

The elements distribution of the FePt and FePtCu NPs was further investigated by STEM-EDS elemental mapping. As shown in Figure 4, Fe (red), Pt (yellow), and Cu (blue) elements are uniformly distributed over the particles, which demonstrates an alloyed nanostructure. There are no rich domains of Fe, Pt or Cu in the samples.

**Figure 5:**
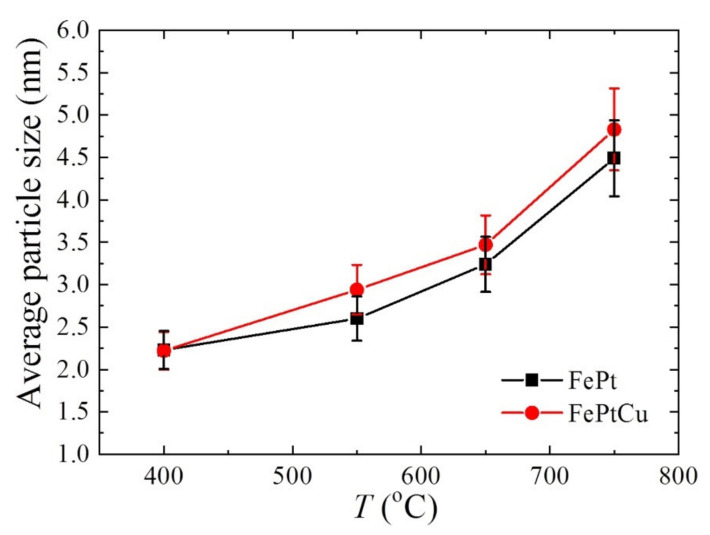
Average particle size (*D*) of FePt and FePtCu nanoparticles at different annealing temperatures.

The average particle sizes (*D*) of FePt and FePtCu NPs are shown in Figure 5. For both FePt and FePtCu NPs, with the increasement of annealing temperature, *D* increases and the size distribution range becomes wider. It is clear that the *D* of FePtCu NPs are larger than that of FePt NPs, which means that adding Cu can increase the particle size of FePt NPs. The *D* of FePt and FePtCu NPs annealed at 400 °C are 2.23 and 2.22 nm, respectively. The size distribution of these two samples ranges from 0.50 to 4.50 nm, and over 90% of the particles are smaller than 3 nm. *D* of FePt NPs annealed at 550 °C slightly increase to 2.60, and a small number of large size particles (6–7 nm) appear. Compared with FePt NPs, FePtCu NPs annealed at 550 °C have larger *D* and the amount of particles with the particle size larger than 3 nm is much larger than that of FePt NPs. When the temperature rises to 650 °C, the *D* of FePt and FePtCu NPs increase to 3.24 and 3.47, respectively. When the annealing temperature further increases to 750 °C, the *D* of FePt and FePtCu NPs increase to 4.49 and 4.83, respectively. The size distribution of samples annealed at 750 °C ranges from 2 to 9 nm, which is much wider than that of samples annealed at lower temperatures. There are also some particles smaller than 3 nm and some particles with the size around 9 nm appearing.

**Figure 6:**
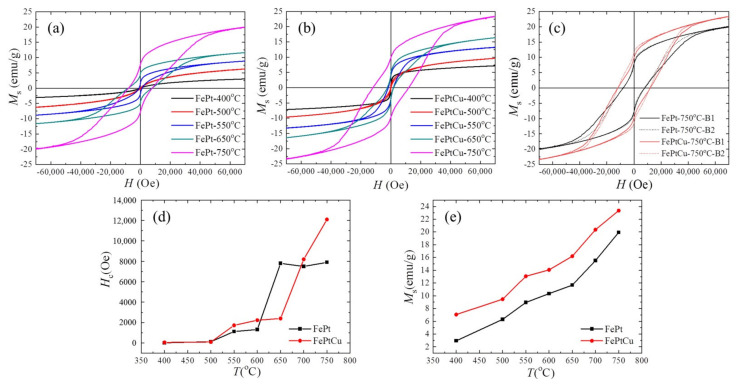
Magnetic hysteresis (M–H) loops of (**a**) FePt and (**b**) FePtCu NPs, (**c**) overlap of the M-H loops of FePt and FePtCu annealed at 750 °C (dashed lines show the other batch of samples), (**d**) the coercivity (*H*_c_) and (**e**) saturation magnetization (*M*_s_) of FePt and FePtCu NPs at different annealing temperatures.

The magnetic hysteresis (M-H) loops were measured by PPMS at 300 K in order to find out how the annealing temperature influences the magnetic properties of the NPs. Figure 6 shows some typical M-H loops of (a) FePt and (b) FePtCu NPs, (c) overlap of the M-H loops of FePt and FePtCu annealed at 750 °C, and (d) the coercivity (*H*_c_) and (e) saturation magnetization (*M*_s_) of FePt and FePtCu NPs at different annealing temperatures. It is shown that the *M*_s_ and *H*_c_ of FePt and FePtCu NPs annealed at 400 °C are very small. The magnetic properties of FePt NPs are related to the *S* and morphology of NPs. FePt and FePtCu NPs annealed at 400 °C are the fcc phase, which indicates low *K*_u_ and small particle size (2.22 nm). The *H*_c_ and *M*_s_ of FePtCu NPs annealed at 550 °C are 1730 Oe and 13.07 emu/g, respectively, which are much larger than those of FePt NPs. A total of 82% of FePt NPs annealed at 550 °C are smaller than 3 nm, which essentially make no contribution to *M*_s_ at 300 K (room temperature). The saturation magnetization of each particle (*m*_s_) is highly volume-dependent because it arises from the collective interaction of atomic magnetic dipoles. As mentioned above, adding Cu can increase the order degree and particle size of FePt NPs. FePtCu NPs annealed at 550 °C have a higher order degree and 39% of the particles are larger than 3 nm, which is twice as large as that of FePt NPs. This is the reason that FePtCu NPs annealed at 550 °C show larger *M*_s_ than that of FePt NPs. As shown in Figure 6e, when the annealing temperature increases, the *M*_s_ of FePt and FePtCu NPs increases. This is because the *S* and *D* of FePt and FePtCu NPs increase as the annealing temperature increases, and, correspondingly, the *M*_s_ of FePt and FePtCu NPs also increases. FePtCu NPs show larger *H*_c_ and higher *M*_s_ than those of FePt NPs due to the fact that adding Cu can improve *S* and *D* of NPs. From TEM images, all the observed NPs are single crystal. According to the Stoner-Wohlfarth model [29], for each single crystal particle, *H*_c_ = *H*_k_ = 2*K*_u_/*m*_s_, where *H*_k_ is the effective anisotropic field. Thus, *H*_c_ of FePt and FePtCu NPs depends on *K*_u_ versus *m*_s_, *K*_u_ is dependent on *S* of the particles, and *m*_s_ is dependent on the volume of particles. As shown in Figure 6d, FePtCu NPs have larger *H*_c_ than that of FePt NPs (except annealing at 650 °C), which indicates that FePtCu NPs have much larger *K*_u_ than that of FePt NPs, which means that adding Cu can greatly improve *K*_u_ of FePt NPs. Figure 6c shows the overlap of M-H loops of FePt and FePtCu annealed at 750 °C; solid lines show one batch (B1) of samples while dashed lines show another batch (B2) of samples. The *H*_c_ and *M*_s_ of FePtCu NPs annealed at 750 °C can reach 12,200 Oe and 23.38 emu/g, respectively. It should be noticed that the M-H loops of FePt and FePtCu NPs annealed at 750 °C show a step where complex magnetic states appeared. This is caused by the wide size of the distribution range of FePt and FePtCu NPs annealed at 750 °C. The *H*_c_ and *M*_s_ of FePtCu NPs annealed at 750 °C are not as high as expected even though *S* is high (0.91). This is because FePtCu NPs are single crystal with *D* of 4.83 nm and some of the particles are smaller than 3 nm. The shapes of the M-H curves of FePt and FePtCu NPs were different, this might be caused by the differences of the size-dispersion and *S* between FePt and FePtCu NPs. Both FePt and FePtCu NPs prepared at different batches annealed at 750 °C have similar shapes and the same *M*_s_ and *H*_c_, which means good reproducibility between them.

The formation mechanism of the FePtCu NPs is shown as follows: Thermal decomposition of Fe (acac)_3_ and Pt (acac)_2_ occurs around 200 °C. At the same time, CuO is reduced to Cu by H_2_ gas. Fe, Pt, and Cu atoms nucleate and form NPs with a chemically disordered fcc structure. The driving forces for forming NPs are likely the Brownian motion and Van der Waals attraction. At this stage, Cu atoms are distributed inside the FePt lattice, which increases the atomic diffusivity and enhances the kinetics of ordering [30]. The reactions involved are shown as follows:

Pt (C_10_H_14_O_4_) → Pt + H_2_O + C

Fe (C_15_H_21_O_6_) → Fe + H_2_O + C

CuO + H_2_ → Cu + H_2_O

No harmful products are present, and no organic solvents, surfactants, chelators, and catalysts are required.

## 4. Conclusions

A simple, green, one-step solid-phase reduction method was proposed to fabricate *L*1_0_-ordered FePt and FePtCu NPs. Fe (acac)_3_, Pt (acac)_2_ and CuO as the precursors were dispersed in NaCl and annealed at 400, 550, 600, 650, 700, and 750 °C in an H_2_-Ar mixed atmosphere. The particles had good dispersion and no sintering or coalescence was observed. All the observed NPs are single crystal. When the annealing temperature increased, *S*, *D*, *H*_c_, and *M*_s_ of FePt and FePtCu NPs increased and the size distribution of the particles became wider. Adding 5% Cu could greatly improve the magnetic properties and morphology of FePt NPs, which was ascribed to Cu atoms distributed inside the FePt lattice, increasing the atomic diffusivity and enhancing the kinetics of ordering. The highest *S*, *D*, *H*_c_, and *M*_s_ could be obtained when FePtCu nanoparticles were annealed at 750 °C, which were 0.91, 4.87 nm, 12,200 Oe, and 23.38 emu/g, respectively. The samples prepared by the current method were too polydisperse, which might hinder their applications. The size dispersion and magnetic properties of FePt NPs could be improved by using a proper weight ratio of the precursors and salt (fixed at 1:1000 in this work). If the precursors (Fe (acac)_3_ and Pt (acac)_2_) dissolved in acetone, it would improve the mixing uniformity of precursors, which might help to improve the size dispersion and magnetic properties of FePt NPs.

## Data Availability

Data sharing not applicable.
